# On the measurement of skeletal muscle anisotropic permittivity property with a single cross-shaped needle insertion

**DOI:** 10.1038/s41598-022-12289-z

**Published:** 2022-05-19

**Authors:** Hyeuknam Kwon, Hyoung Churl Park, Albert Cheto Barrera, Seward B. Rutkove, Benjamin Sanchez

**Affiliations:** 1 Division of Software, Yonsei University, Wonju, Republic of Korea; 2 Department of Mathematics, Yonsei University, Wonju, Republic of Korea; 3 Department of Electrical and Computer Engineering, University of Utah, Salt Lake City, UT 84112 USA; 4 Department of Neurology, Beth Israel Deaconess Medical Center, Harvard Medical School, Boston, MA 02215 USA

**Keywords:** Biophysics, Characterization and analytical techniques, Biomarkers

## Abstract

Application of minimally invasive methods to enable the measurement of tissue permittivity in the neuromuscular clinic remain elusive. This paper provides a theoretical and modeling study on the measurement of the permittivity of two-dimensional anisotropic tissues such as skeletal muscle with a multi-electrode cross-shaped needle. For this, we design a novel cross-shaped needle with multiple-electrodes and analyse apparent impedance corresponding to the measured impedance. In addition, we propose three methods of estimate anisotropic muscle permittivity. Compared to existing electrical impedance-based needle methods that we have developed, the new needle design and numerical methods associated enable estimating in vivo muscle permittivity values with only a single needle insertion. Being able to measure muscle permittivity directly with a single needle insertion could open up an entirely new area of research with direct clinical application, including using these values to assist in neuromuscular diagnosis and to assess subtle effects of therapeutic intervention on muscle health.

## Introduction

Understanding the basic characteristics of electrical current flow through muscle is needed for improving the accuracy of existing analytical techniques, such as needle electromyography, as well as developing new diagnostic tools for neuromuscular disorder (NMD) assessment. For example, volume conduction theory explains that high frequency electrical events generated during myofiber depolarization are visible only when the recording electrodes are in close proximity to the myofiber source, whereas low frequency events attenuate less and can propagate through the extracellular space over a long distance^1–3^. This low-pass filtering characteristics affects the morphology (i.e., in amplitude and latency) of extracellular local field potentials in muscle tissue. These changes are likely the result of complex interactions of many NMD-related tissue alterations affecting the extracellular conducting medium (e.g., myofiber atrophy and fatty infiltration in dystrophic muscle)^4,5^. Although these underlying alterations directly affect the microenvironment’s passive, linear permittivity property (i.e., conductivity and relative permittivity) as well as their spatial dependence and are a main determinant of the frequency-filtering properties of electrical potential distribution within diseased muscle, evaluation of these changes using standard needle electromyography (EMG) is not possible^6^.

Methods for measuring the muscle permittivity have limitations. Magnetic resonance electrical properties (MR-EP) imaging can provide noninvasive imaging of tissue permittivity; however, it has been mainly applied to the central nervous system^7^. Also, the technique requires subjects to go to a specialized facility, it cannot be used in children without sedation, subjects must lie flat—a major problem in people with respiratory compromise, a common situation in patients with NMD—, it can put subjects with implanted pacemakers at risk, it is very expensive to perform, and the test is slow, typically taking 45 min or longer to perform. Perhaps most importantly, MR-EP imaging can only assess tissue permittivity at a single frequency determined by the strength of the magnetic field (typically >100 MHz) and thus these electrical properties have limited value to aid in the interpretation of EMG data, which is typically in the Hz to kHz frequency range.

The gold standard electrical impedance-based method for measuring muscle permittivity is muscle biopsy^8–10^, in which a sample of freshly excised muscle is studied. However, due to the invasiveness^11,12^, researchers have mainly obtained ex vivo values from laboratory animals. In addition to the limitations in recapitulating the physiopathology of human NMDs, ex vivo animal muscle values differ from in vivo human tissue as these properties change, among others, within animal species^13–16^, with death^17,18^ and with temperature^19^. Another major limitation of biopsy is the inability to follow the natural progression or remission of NMDs over time because of sampling limitations.

To allow the measurement of in vivo muscle permittivity values^20,21^, we developed needle electrical impedance myography (EIM) and associated methods^22,23^. While promising, these methods required multiple simultaneous needle insertions. Thus, it became clear that further research was still needed to facilitate clinical adoption by minimizing patient discomfort to only a single needle insertion^24^. Here, we present an innovative cross-shaped EIM needle and associated (iterative) inverse methods for estimating in vivo muscle permittivity with only a single needle insertion.

The rest of the text consists of three sections; “Methods”, “Results”, and “Discussion”. In the “Methods” section, we define needle model and muscle domain model and derive the equation of apparent electrical impedance of using the needle in the muscle model (in the subsection “Forward problem”). Based on the apparent electrical impedance equation, three methods for estimating the anisotropic permittivity properties of muscles are introduced in the inverse methods subsection (in the subsection “inverse methods”). Finally, this section describes the setting up of numerical experiments to verify these methods (in the subsection “Numerical simulations”). The results section provides numerical simulation results applying the three methods. Numerical simulations are designed to validate Convergence, Sensitivity, and Robustness. In addition, the results of estimating the electrical properties of the anisotropic muscle are also presented. The final section of the paper is the “Discussion” section.

## Methods

Table 1 summarizes the relevant nomenclature, including symbols and parameters, that is used throughout the manuscript.

**Table 1:** Symbols and parameters used in this study.

Symbol	Unit	Description
$\theta$	Radian	Needle azimutal angle, set to be 0 in this paper
$\varphi$	Radian	Needle polar angle, set to be 0 in this paper
$\phi$	Radian	Needle rotation angle, set to be positive number in this paper
*a*	m	Distance between the first and second rows of the electrode array (= third and fourth rows)
*b*	m	Distance between the second and third rows of the electrode array
*s*	m	Distance between two columns of electrode array
$\kappa _{\mathrm{L}}$ and $\kappa _{\mathrm{T}}$	$\Omega$ m	Longitudinal and transverse impedivity
${\bar{\kappa }}$	$\Omega$ m	Geometric mean of the impedivity, defined as ${\bar{\kappa }}:=\sqrt{\kappa _{ \text {L}}\kappa _{\text {T}}}$
$\rho _{\mathrm{L}}$ and $\rho _{\mathrm{T}}$	$\Omega$ m	Longitudinal and transverse resistivity
$\tau _{\mathrm{L}}$ and $\tau _{\mathrm{T}}$	$\Omega$ m	Longitudinal and transverse reactivity
$\alpha ^2$	Dimensionless	Anisotropy ratio of the muscle, defined as $\rho _{\mathrm{L}}/\rho _{\mathrm{T}}$
$K_{\mathrm{half\,space}}$	Dimensionless	Domain factor of the half space, that is $2\pi$
${\widehat{K}}_i$ and ${\widehat{K}}_i^\perp$	Dimensionless	Domain factor of the needle for $i=1,2,4,6,17$
$Z_i$ and ${\widehat{Z}}_i$	$\Omega$	Measured and simulated apparent impedance for $i=1,2,4,6,17$

### Forward problem

#### Needle and muscle domain models

**Figure 1:**
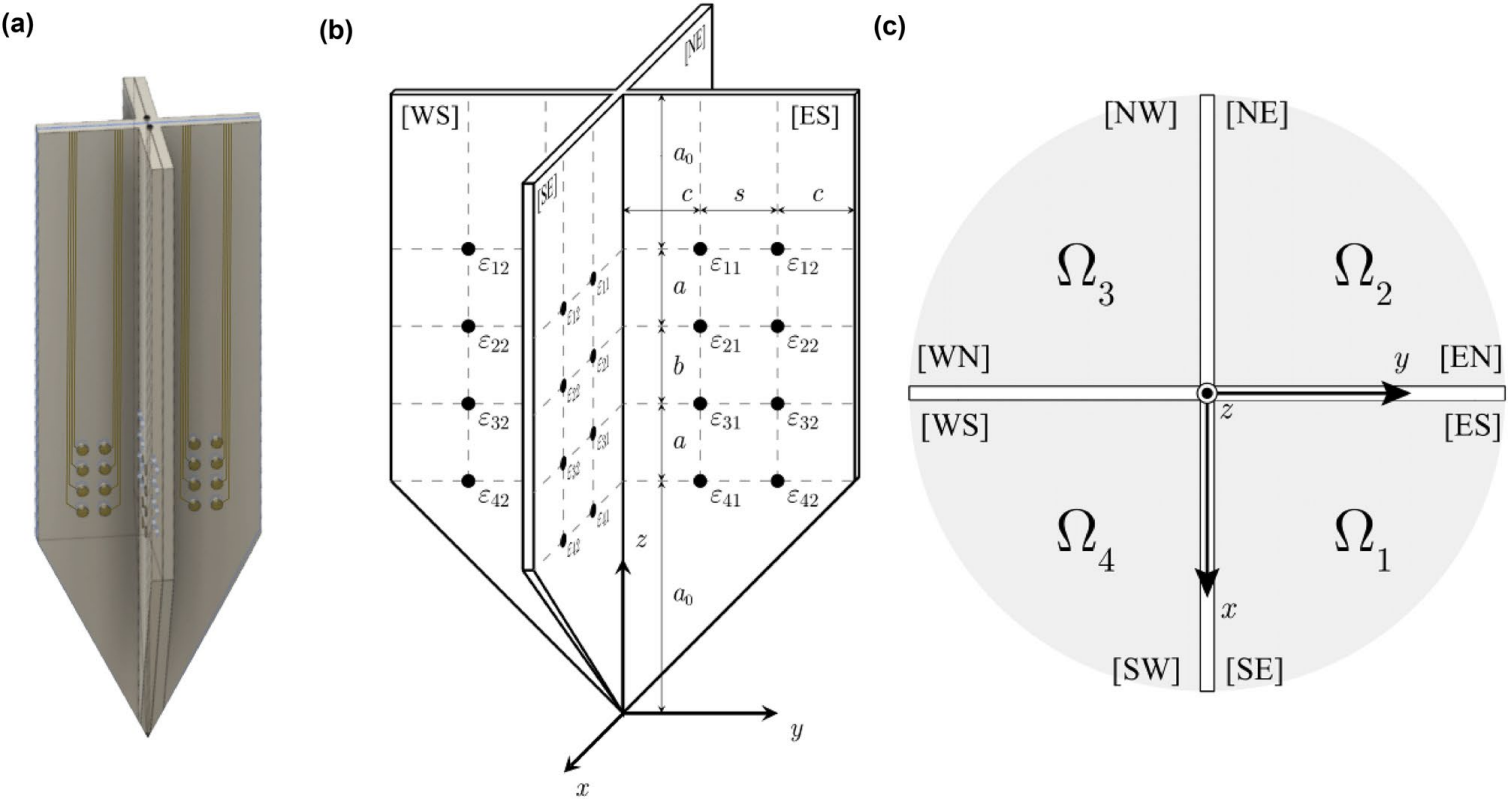
**(a)** Schematic representation of the needle. **(b)** Needle’s geometry (not to scale). **(c)** Needle’s top view.

We propose a cross-type ‘+’ needle shown in Fig. 1a containing 64 point-like electrodes distributed in the eight faces of the needle (Fig. 1b), each face referred as to $[F_1F_2]$ where $F_1$ and $F_2$ denote the face’s cardinal point and orientation respectively, i.e., $F_1,F_2\in \{N,S,W,E\}$, so that when $F_1 \in \{N,S\}$ then $F_2 \in \{E,W\}$ and when $F_1 \in \{E,W\}$ then $F_2 \in \{N,S\}$ as in Fig. 1b. Each face has eight electrodes $\varepsilon _{ik}$ distributed in a four rows $\times$ two columns, where $i=1,2,3,4$ is the row index and $k=1,2$ is the column index. The distance *a* (m) is defined between the outermost electrodes and the nearest electrodes, i.e., $\varepsilon _{1k}$ to $\varepsilon _{2k}$ and $\varepsilon _{3k}$ to $\varepsilon _{4k}$, while the distance *b* (m) is defined between the inner electrodes $\varepsilon _{2k}$ to $\varepsilon _{3k}$. The distance from both the needle tip and the needle’s upper edge to the nearest electrodes $\varepsilon _{1k}$ and $\varepsilon _{4k}$ is $a_0$ (m), respectively. The distance between $\varepsilon _{i1}$ and $\varepsilon _{i2}$ is *s* (m). Finally, both distances from $\varepsilon _{i1}$ to the needle’s major axis and from $\varepsilon _{i2}$ to the lateral edge is *c* (m). We then assume that the four arms of the needle define four semi-infinite subdomains in Fig. 1c, namely $\Omega _{1}=\{x>0,y>0,z\in {\mathbb {R}} \}$, $\Omega _{2}=\{x<0,y>0,z\in {\mathbb {R}} \}$, $\Omega _{3}=\{x<0,y<0,z\in {\mathbb {R}} \}$ and $\Omega _{4}=\{x>0,y<0,z\in {\mathbb {R}} \}$. In other words, the needle arms’ thickness is considered negligible.

**Figure 2:**
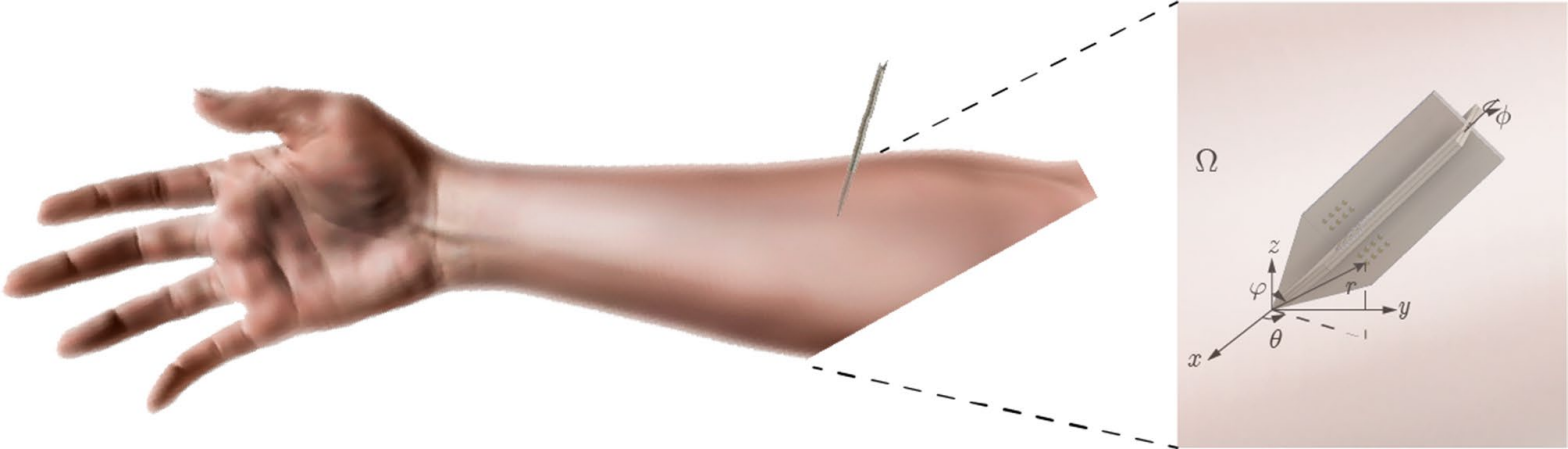
Insertion of the needle to the skeletal muscle and inlet representing a model abstraction of an arbitrary geometrical arrangement of muscle domain $\Omega$ and needle to illustrate the spherical coordinate system used.

The needle is inserted through the skin and subcutaneous fat tissues into the muscle as shown in Figure 2. We define the *x*, *y*-axes transverse to the longitudinal direction determined by the muscle fibers in the *z*-axis. They are defined to form the canonical basis in ${\mathbb {R}}^3$ and for the needle’s major axis to be located in the first quadrant of the *yz* plane. We use spherical coordinates to define the position of the needle’s major axis, with the origin of coordinates defined by convention located in the needle’s tip. The polar angle is defined by $\varphi \in [0,\pi /2]$. By definition of the axes the azimutal angle $\theta$ is always 0. The rotation angle $\phi \in [0,2\pi )$ defines the needle’s rotation angle with respect to its major axis.

As a convention, when $\theta = \varphi = \phi = 0$, then the needle’s major axis is aligned with respect to the *z*-axis and the [ES] face is the *yz* plane. Finally, we define when $\varphi = \pi /2$ then $\phi \in [0,\pi )$.

#### Electrodes’ position in spherical coordinates

Unless otherwise noted, henceforth we restrict ourselves to study an impedance measurement with the electrodes in the face [ES]. The position $\mathbf{P }_{ik}:=(x_{ik},y_{ik},z_{ik}) \in {\mathbb {R}}^3$ of electrode $\varepsilon _{ik}$ by default, i.e., $\varphi = \phi = \theta = 0$, is

1 $$\begin{array}{rclcrcl} \mathbf{P }_{11} &{}=&{} (0,c,2a+b+a_0) &{}  \&  &{} \mathbf{P }_{12} &{}=&{} (0,c+s,2a+b+a_0) \\ \mathbf{P }_{21} &{}=&{} (0,c,a+b+a_0) &{}  \&  &{} \mathbf{P }_{22} &{}=&{} (0,c+s,a+b+a_0) \\ \mathbf{P }_{31} &{}=&{} (0,c,a+a_0) &{}  \&  &{} \mathbf{P }_{32} &{}=&{} (0,c+s,a+a_0) \\ \mathbf{P }_{41} &{}=&{} (0,c,a_0) &{}  \&  &{} \mathbf{P }_{42} &{}=&{} (0,c+s,a_0) \end{array}$$

as we see in Fig. 1b. For notational simplicity, we denote the electrode position by $(0,y_{ik},z_{ik}):=\mathbf{P }_{ik}$ for $i=1,2,3,4$ and $k=1,2$. To describe the general position of an electrode we use three dimensional rotation matrices. The rotation matrices along *x*-, *y*-, and *z*- axes with an angle $\delta$ using right-hand rule are

2 $$\begin{aligned}  R_{x}(\delta ) := \begin{bmatrix} 1&{}0&{}0 \\ 0 &{} \cos \delta &{} -\sin \delta \\ 0 &{} \sin \delta &{} \cos \delta \\ \end{bmatrix}, \quad \qquad { R_{y}(\delta ) := \begin{bmatrix} \cos \delta &{} 0 &{} -\sin \delta \\ 0&{}1&{}0 \\ \sin \delta &{} 0 &{} \cos \delta \\ \end{bmatrix} } \qquad \text {and} \quad R_{z}(\delta ) := \begin{bmatrix} \cos \delta &{} -\sin \delta &{} 0 \\ \sin \delta &{} \cos \delta &{} 0 \\ 0&{}0&{}1 \\ \end{bmatrix}. \end{aligned}$$

The rotated electrode position $\mathbf{Q }_{ik} \in {\mathbb {R}}^3$ is defined as $\mathbf{Q }_{ik}$ := $R_x(-\varphi )R_z(\phi )\mathbf{P }_{ik}$, that is

3 $$\begin{aligned} \mathbf{Q }_{ik} = \begin{bmatrix} -y_{ik}\sin \phi \\ y_{ik}\cos \phi \cos \varphi +z_{ik}\sin \varphi \\ -y_{ik}\cos \phi \sin \varphi +z_{ik}\cos \varphi \end{bmatrix} =: \begin{bmatrix} \mathbf{Q }_{ik}(x)\\ \mathbf{Q }_{ik}(y) \\ \mathbf{Q }_{ik}(z) \end{bmatrix}. \end{aligned}$$

If we want to describe the position of an electrode in another face it is equivalent to consider the electrode to be in the [ES] face changing the angle $\phi$ for $\phi + t\pi /2$ with $t \in {\mathbb {Z}}$.

#### Apparent electrical impedance

We define the impedivity $\kappa$ ($\Omega$ m) $\in {\mathbb {C}}$ as

4 $$\kappa := \rho + j\tau = \frac{\sigma }{\sigma ^2 + (\omega \epsilon _0\epsilon _\text {r})^2}-j\frac{\omega \epsilon _0\epsilon _\text {r}}{\sigma ^2 + (\omega \epsilon _0\epsilon _\text {r})^2},$$

where $\rho$ and $\tau$ are the resistivity and reactivity, respectively, $j^2 = -1$ is the imaginary unit (dimensionless), $\sigma$ is the conductivity (S $\hbox {m}^{-1}$), $\omega$ is the angular frequency (rad $\hbox {s}^{-1}$), $\epsilon _0$ is the vacuum permittivity (F $\hbox {m}^{-1}$) and $\epsilon _\text {r}$ is the relative permittivity (dimensionless). We note that if $\epsilon _\text {r} = 0$, then $\kappa = \rho = 1/\sigma$.

Let $\alpha ^2 := \frac{\rho _{\text {L}}}{\rho _\text {T}} := \frac{\tau _\text {L}}{\tau _\text {T}} \le 1$ be the anisotropy ratio (dimensionless), where the subscripts {T,L} denote the transverse *x*,*z*-axes and longitudinal *y*-axis directions. Following Plonsey and Heppner^33^ and Kwon et al.^20^ work, the potential *V* (V) $\in {\mathbb {C}}$ created by a point current electrode in an homogeneous infinite anisotropic material is

5 $$V :=\frac{{\bar{\kappa }}I}{K \left\Vert \mathbf{r }_\alpha - \mathbf{r }_0\right\Vert _2} =\frac{{\bar{\kappa }}I}{K \sqrt{(x-x_0)^2+\alpha ^2(y-y_0)^2+(z-z_0)^2}},$$

where ${\bar{\kappa }}:=\sqrt{\kappa _{ \text {L}}\kappa _{\text {T}}}$ is the geometric mean of the longitudinal and transverse impedivities, *I* is the current (A), $K := 2\pi$ is a domain factor (dimensionless) from assuming that the needle face delimits the muscle in a semi-infinite region^34^. The operator $\left\Vert \cdot \right\Vert _2$ is the $L_2$ norm, $\mathbf{r }_{0}:=(x_0, \alpha y_0, z_0)$ and $\mathbf{r }_{\alpha }:=(x, \alpha y, z)$ are the apparent position of current and voltage electrodes, respectively.

For notational convenience, we define the apparent distance $d_{\alpha }$ m between two electrodes (${\mathbf {Q}}_{ik}$, ${\mathbf {Q}}_{nl}$) as

6 $$\begin{aligned}  d_\alpha ({\mathbf {Q}}_{ik},{\mathbf {Q}}_{nl}) := \sqrt{({\mathbf {Q}}_{ik}(x) - {\mathbf {Q}}_{nl}(x))^2 + \alpha ^2({\mathbf {Q}}_{ik}(y) - {\mathbf {Q}}_{nl}(y))^2 + ({\mathbf {Q}}_{ik}(z) - {\mathbf {Q}}_{nl}(z))^2}. \end{aligned}$$

**Figure 3:**
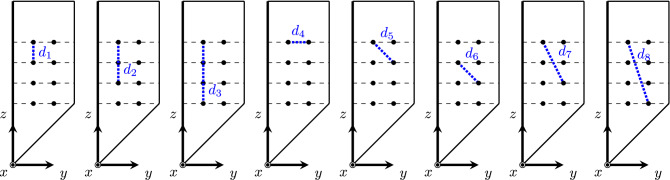
The eight different distances between two same face electrodes. Face [ES] in default side shown.

Using the expression above and the position of electrodes in Eq. (3) we obtain

7 $$\begin{aligned} d_\alpha ({\mathbf {Q}}_{ik},{\mathbf {Q}}_{nl}) = \sqrt{(y_{ik} - y_{nl})^2A + (z_{ik} - z_{nl})^2 B + (y_{ik} - y_{nl})(z_{ik} - z_{nl})C}, \end{aligned}$$

where

8 $$\begin{aligned} A &:= \sin ^2\phi + \cos ^2\phi (\alpha ^2\cos ^2\varphi + \sin ^2\varphi ), \\B &:= 1, \\C &:= 2(\alpha ^2 - 1)\cos \phi \sin \varphi \cos \varphi = (\alpha ^2 - 1)\cos \phi \sin 2\varphi , \end{aligned} $$

are variables that only depend on the needle orientation and the anisotropy ratio. As expected, when $\alpha ^2 = 1$ (i.e., isotropic muscle) the dependence of $d_\alpha$ with the orientation of the needle is lost. If we choose two electrodes on the same face, there are eight different distances considering the electrodes’ distribution (see Fig. 3). We define them as $d_{1,...,8}$

9 $$\begin{aligned} {} &d_1 := d_\alpha ({\mathbf {Q}}_{in},{\mathbf {Q}}_{(i+1)n}) = a\sqrt{B}, i\in \{1,3\}, n \in \{1,2\},\\&d_2 := d_\alpha ({\mathbf {Q}}_{in},{\mathbf {Q}}_{(i+2)n}) = (a + b)\sqrt{B}, i\in \{1,2\}, n \in \{1,2\},\\&d_3 := d_\alpha ({\mathbf {Q}}_{1n},{\mathbf {Q}}_{4n}) = (2a + b)\sqrt{B}, n \in \{1,2\},\\&d_4 := d_\alpha ({\mathbf {Q}}_{i1},{\mathbf {Q}}_{i2}) = s\sqrt{A}, i\in \{1,2,3,4\},\\&d_5 := d_\alpha ({\mathbf {Q}}_{in},{\mathbf {Q}}_{(i+1)k}) = \sqrt{s^2A + a^2B + saC}, i\in \{1,3\}, \{n,k\} = \{1,2\},\\&d_6 := d_\alpha ({\mathbf {Q}}_{2n},{\mathbf {Q}}_{3k}) = \sqrt{s^2A + b^2B + sbC}, \{n,k\} = \{1,2\},\\&d_7 := d_\alpha ({\mathbf {Q}}_{in},{\mathbf {Q}}_{(i+2)k})= \sqrt{s^2A + (a + b)^2B + s(a + b)C}, i\in \{1,2\}, \{n,k\} = \{1,2\},\\&d_8 := d_\alpha ({\mathbf {Q}}_{1n},{\mathbf {Q}}_{4k})= \sqrt{s^2A + (2a + b)^2B + s(2a + b)C}, \{n,k\} = \{1,2\}.\end{aligned}$$

To perform an impedance measurement, we need four electrodes being positive and negative current and voltage electrodes which are denoted by $\varepsilon _{I^{+}}$, $\varepsilon _{I^{-}}$, $\varepsilon _{V^{+}}$ and $\varepsilon _{V^-}$, respectively. Their positions are denoted by ${\mathbf {Q}}_{I^+}$, ${\mathbf {Q}}_{I^-}$, ${\mathbf {Q}}_{V^+}$ and ${\mathbf {Q}}_{V^-}$. Considering Eqs. (5) and (7), we can rewrite electrical potential as follows

10 $$\begin{aligned} V^{pm} = \frac{{{\bar{\kappa }}}I}{Kd_\alpha ({\mathbf {Q}}_{V^{p}}, {\mathbf {Q}}_{I^{m}})}, \end{aligned}$$

where $p,m \in \{+,-\}$ and $V^{pm}$ is the electric potential measured at $\varepsilon _{V^{p}}$ when current *I* generated at $\varepsilon _{I^m}$. Finally, we define the impedance as

11 $$\begin{aligned} Z:=\frac{V^{++}-V^{+-}-V^{-+}+V^{--}}{I} \end{aligned}$$

**Figure 4:**
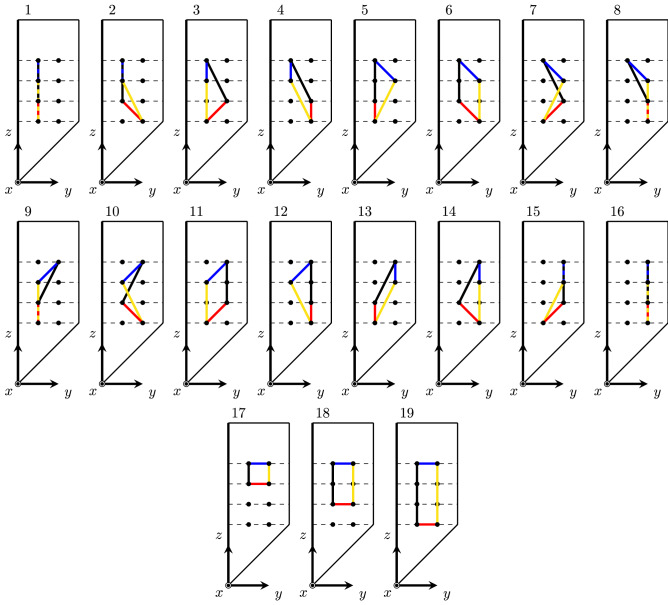
Configurations 1 to 19. In blue, distance for $V^{++}$. In red, distance for $V^{--}$. In yellow, distance for $V^{+-}$. In black, distance for $V^{-+}$.

To measure the impedance using the four electrode method, we will have to measure the distance between two electrode roles instead of two electrodes. Therefore, $d_\alpha (\cdot ,\cdot )$ in Eq. (10) is presented like so. If we choose four electrodes on the same face for an impedance measurement, i.e., $\varepsilon _{I^{+}}$, $\varepsilon _{I^{-}}$, $\varepsilon _{V^{+}}$ and $\varepsilon _{V^-}$, there are $\genfrac(){0.0pt}1{8}{4} \cdot 4! = 1680$ possibilities. Most of the configurations give redundant information so we restrict ourselves to nineteen configurations (see Fig. 4). Configurations 1 to 16 in Fig. 4 verify $\{\varepsilon _{11},\varepsilon _{12}\} \in \varepsilon _{I^{+}}$, $\{\varepsilon _{41},\varepsilon _{42}\} \in \varepsilon _{I^{-}}$, $\{\varepsilon _{21},\varepsilon _{22}\} \in \varepsilon _{V^{+}}$ and $\{\varepsilon _{31},\varepsilon _{32}\} \in \varepsilon _{V^{-}}$. Configurations 17 to 19 are rectangular configurations. The expressions of the impedance for each configuration in Fig. 4 are presented in Table 2. Note that two configurations that have the sets $\{d_\alpha ({\mathbf {Q}}_{V^{+}}, {\mathbf {Q}}_{I^{+}}), d_\alpha ({\mathbf {Q}}_{V^{-}}, {\mathbf {Q}}_{I^{-}})\}$ and $\{d_\alpha ({\mathbf {Q}}_{V^{+}}, {\mathbf {Q}}_{I^{-}}), d_\alpha ({\mathbf {Q}}_{V^{-}}, {\mathbf {Q}}_{I^{+}})\}$ equal give the same impedance value as shown in Table 2.

**Table 2:** Table of the nineteen configurations in Fig. 4 with its impedance equation (11) and the electrode involved for each role.

Configurations	Impedance	${\mathbf {Q}}{}$ subscript			
		${I^+}$	${V^+}$	${V^-}$	${I^-}$
1	$Z_1 = \frac{2{\bar{\kappa }}}{K}\left( \frac{1}{d_1}- \frac{1}{d_2}\right)$	11	21	31	41
2	$Z_2 = \frac{{\bar{\kappa }}}{K}\left( \frac{1}{d_1}-\frac{1}{d_7}-\frac{1}{d_2}+\frac{1}{d_5}\right)$	11	21	31	42
3	$Z_3 = Z_2$	11	21	32	41
4	$Z_4 = \frac{2{\bar{\kappa }}}{K}\left( \frac{1}{d_1}- \frac{1}{d_7}\right)$	11	21	32	42
5	$Z_5 = Z_2$	11	22	31	41
6	$Z_6 = \frac{2{\bar{\kappa }}}{K}\left( \frac{1}{d_5}- \frac{1}{d_2}\right)$	11	22	31	42
7	$Z_7 = \frac{2{\bar{\kappa }}}{K}\left( \frac{1}{d_5}- \frac{1}{d_7}\right)$	11	22	32	41
8	$Z_8 = Z_2$	11	22	32	42
9	$Z_9 = Z_2$	12	21	31	41
10	$Z_{10} = Z_7$	12	21	31	42
11	$Z_{11} = Z_6$	12	21	32	41
12	$Z_{12} = Z_2$	12	21	32	42
13	$Z_{13} = Z_4$	12	22	31	41
14	$Z_{14} = Z_2$	12	22	31	42
15	$Z_{15} = Z_2$	12	22	32	41
16	$Z_{16} = Z_1$	12	22	32	42
17	$Z_{17} = \frac{2{\bar{\kappa }}}{K}\left( \frac{1}{d_4}- \frac{1}{d_1}\right)$	11	12	21	22
18	$Z_{18} = \frac{2{\bar{\kappa }}}{K}\left( \frac{1}{d_4}- \frac{1}{d_2}\right)$	11	12	31	32
19	$Z_{19} = \frac{2{\bar{\kappa }}}{K}\left( \frac{1}{d_4}- \frac{1}{d_3}\right)$	11	12	41	42

In this paper, we choose to use five different current-voltage configurations (1, 2, 4, 6, 17 in Fig. 4) to measure apparent impedance *Z*. Using Eqs. (5) and (11), the impedance values are

12 $$\begin{aligned} Z_1= & {} \frac{{\bar{\kappa }}}{K}\frac{2b}{a(a+b)} \end{aligned}$$

13 $$\begin{aligned} Z_2= & {} \frac{{\bar{\kappa }}}{K}\left( \frac{1}{a\sqrt{B}} - \frac{1}{\sqrt{s^2A + (a + b)^2B + s(a + b)C}} - \frac{1}{(a + b)\sqrt{B}} + \frac{1}{\sqrt{s^2A + a^2B + saC}}\right) \end{aligned}$$

14 $$\begin{aligned} Z_4= & {} \frac{2{\bar{\kappa }}}{K} \frac{[s^2A + (a+b)^2+(a+b)sC]\sqrt{B}-aB\sqrt{s^2A+(a+b)^2B+(a+b)sC}}{aB[s^2A+(a+b)^2B+(a+b)sC]} \end{aligned}$$

15 $$\begin{aligned} Z_6= & {} \frac{2{\bar{\kappa }}}{K} \frac{(a+b)B\sqrt{s^2A+a^2B+asC} - \sqrt{B}(s^2A+a^2B+asC)}{(s^2A+a^2B+asC)(a+b)B} \end{aligned}$$

16 $$\begin{aligned} Z_{17}= & {} \frac{2{\bar{\kappa }}}{K} \frac{a\sqrt{B} - s\sqrt{A}}{as\sqrt{AB}} \end{aligned}$$

Here, *A*, *B*, and *C* are defined in Eq. (8).

Of note, $Z_i$ with $i=\{1,2,4,6,17\}$ are inversely proportional to the *unknown* geometric factor *K* (dimensionless), the latter dependent on the electrode size and the inter-electrode distances. We propose an empirical needle geometric factor of the needle ${\widehat{K}}_i$ and ${\widehat{K}}_i^\perp$ as

17 $$\begin{aligned} {\widehat{K}}_i(\alpha ^2,\phi ) = K_{\mathrm{half\,space}} \frac{Z_i(\alpha ^2,\phi )}{{\widehat{Z}}_i(\alpha ^2,\phi )} \quad \text{ and }\quad {\widehat{K}}_i^\perp (\alpha ^2,\phi ) = {\widehat{K}}_i(\alpha ^2,\phi -\pi /2) \end{aligned}$$

for given anisotropy ratio $\alpha ^2$ and needle rotation angle $\phi$, where $K_{\mathrm{half\,space}}$ is $2\pi$, $Z_i(\alpha ^2,\phi )$ is the apparent impedance value derived from Eqs. (12)–(16) with half space domain assumption, and ${\widehat{Z}}_i(\alpha ^2,\phi )$ is the simulated apparent impedance value.

### Inverse methods

In this section, we provide three inverse methods to estimate the anisotropic permittivity properties of muscle from apparent impedance values measured using the cross-shaped needle electrodes. Methods are presented in increasing complexity:

1. Method I requires to insert the cross-shaped needle has its major axis is aligned with respect to the *z*-axis and the [ES] face is the *yz*-plane as in Fig. 1 b, i.e. $\varphi = \phi = 0$. Method I can be applied using only electrodes on one side, for example electrodes on [ES].
2. The needle of method II rotated the needle of method I about the *z*-axis by known angle $\phi$, i.e. $\varphi =0$ and $\phi$ don’t need to be zero. Method II can be applied using only electrodes on one side, for example electrodes on [ES].
3. The needle position of method II is same as that of method III, but method III does not require to know the needle rotation angle $\phi$. Method III requires the use of electrodes on two facing sides, for example electrodes on [ES] and [SE].

The key idea of the iteration process is to update the needle geometric factor *K* in Eqs. (12)–(16). We use ${\widehat{K}}_i$ in Eq. (17) to estimate *K*. To do that, we built a dataset of ${\widehat{K}}_i$ values for each needle electrode model $i=1,2,4,6,17$ while changing muscle anisotropy and needle rotation angle in numerical simulations. Indeed, in order to make a dataset of ${\widehat{K}}_i$ with fixed needle electrode model, we perform several numerical simulations with various $\alpha ^2 \in (0,1]$ and $\phi \in [0,\pi /2)$.

#### Inverse Method I: $Z_1$ and $Z_i$ are measured in $\theta =0$ (or $\pi /2$), $\phi =0$, $\varphi =0$ for $i \in \left\{ 2,4,6,17\right\}$

Assumption 1.: The major muscle fibers orientation is known and the needle axis is aligned on the *z*-axis (i.e., the needle is inserted perpendicularly to the muscle fibers).

**Figure 5:**
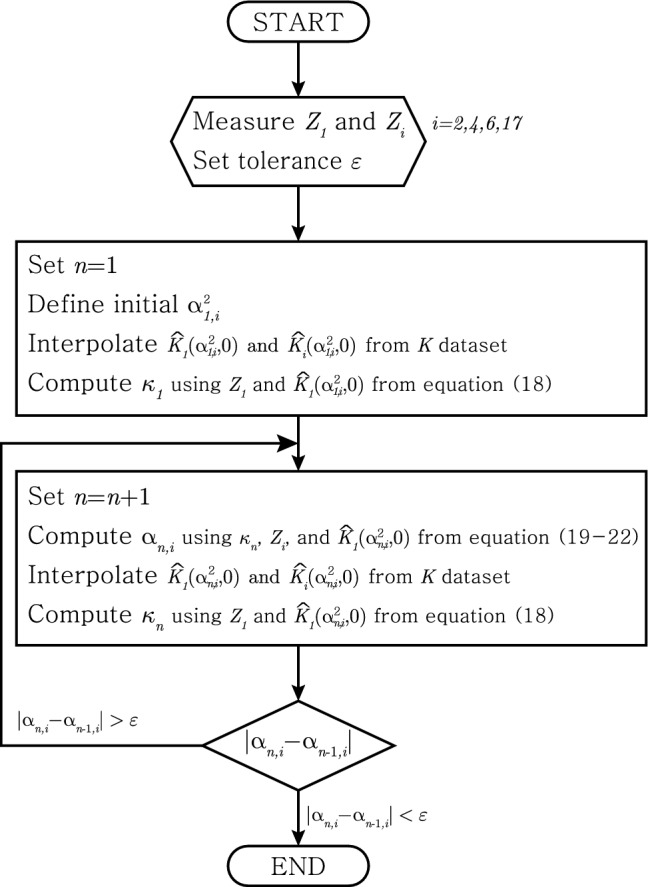
Iteration scheme of the method I.

Method I uses four electrodes on a plane parallel to the direction of the muscle fibre (e.g. [ES] plane in Fig. 1). On the selected plane, we measure two impedances $Z_1$ and $Z_i$ for $i=2,4,6,17$ as in Fig. 4. The measured $Z_1$ is used to compute ${\bar{\kappa }}$ and measured $Z_i$ is used to compute $\alpha ^2$. The flowchart can be seen in Fig. 5.

#### Estimation of ${\bar{\kappa }}$

We estimate ${\bar{\kappa }}$ as a convergence value of $\kappa _{n}$, i.e. ${\bar{\kappa }}:=\lim _{n\rightarrow \infty }\kappa _n$, and define $\kappa _{n}$ as

18 $$\begin{aligned} \kappa _{n} = \frac{a(a+b)Z_{1}K_1(\alpha ^2_{n,i},0)}{2b} \end{aligned}$$

where $\alpha ^2_{n,i}$ is estimated anisotropy ratio at *n*-th iteration for $i=2,4,6,17$ which indicates used impedance value.

#### Estimation of $\alpha ^2$

We estimate $\alpha ^2$ as a convergence value of $\alpha ^2_{n,i}$, i.e. $\alpha ^2:=\lim _{n\rightarrow \infty }\alpha ^2_{n,i}$ for $i=2,4,6,17$. Note here that *n* is for iteration number and *i* is for used impedance number. We propose here four methods to estimate $\alpha ^2_{n,i}$ of using $Z_i$ for $i=2,4,6,17$.

- Using $Z_2$: $\alpha _{n,2}^2$ is the solution of below equation
  19 $$\begin{aligned} Q^4x^4 -4(Q^2+2Q^4R)x^3 -6(Q^2R+Q^4R^2)x^2 -2Q^2R^2 + R^2 =0 \end{aligned}$$
  where $$Q:= \frac{Z_2{\widehat{K}}_2(\alpha _{n-1,2}^2,0)}{\kappa _{n-1}}-\frac{1}{a}+\frac{1}{a+b}$$ and $$R := 2ab+b^2$$.
- Using $$Z_4$$:
  20 $$\begin{aligned} \alpha ^2_{n,4} = \frac{Q-(a+b)^2}{s^2} \end{aligned}$$
  where $$Q := \left( \frac{2a\kappa _{n-1}}{aZ_{4}{\widehat{K}}_4(\alpha _{n-1,4}^2,0)-2\kappa _{n-1}} \right) ^2$$.
- Using $$Z_6$$:
  21 $$\begin{aligned} \alpha ^2_{n,6} = \frac{Q - a^2}{s^2} \end{aligned}$$
  when $$Q := \left( \frac{2a(a+b)\kappa _{n-1}}{(a+b)Z_{6} {\widehat{K}}_6(\alpha _{n-1,6}^2,0)+2\kappa _{n-1}}\right) ^2$$
- Using $$Z_{17}$$:
  22 $$\begin{aligned} \alpha ^2_{n,17} = \left( \frac{2a\kappa _{n-1}}{asZ_{17}{\widehat{K}}_{17}(\alpha _{n-1,17}^2,0)+2\kappa _{n-1})}\right) ^2 \end{aligned}$$

#### Inverse Method II: $Z_1$ and $Z_i$ are measured in $\theta =0$, $\varphi =0$ for $i \in \left\{ 2,4,6,17\right\}$

Assumption 1.: Same as method I
Assumption 2.: Known $$\phi \ne 0$$

**Figure 6:**
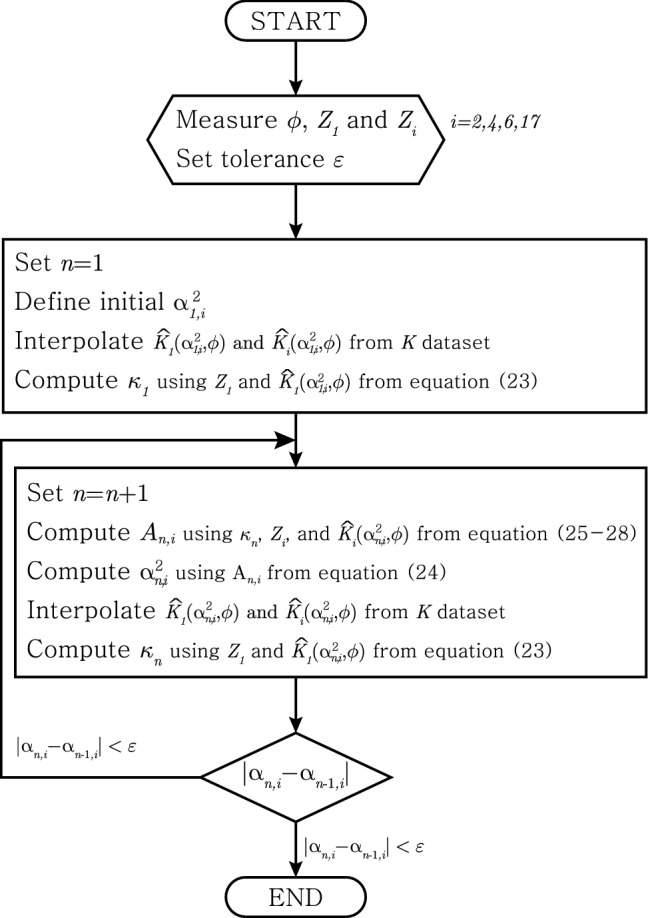
Iteration scheme of the method II.

Method II uses four electrodes on a plane with an angle $\phi$ to the direction of the muscle fibre. On the selected plane, we measure two impedances $Z_1$ and $Z_i$ for $i=2,4,6,17$ as in Fig. 4. The measured $Z_1$ is used to compute ${\bar{\kappa }}$ and measured $Z_i$ is used to compute $\alpha ^2$. The flowchart can be seen in Fig. 6.

#### Estimation of ${{\bar{\kappa }}}$

We estimate ${{\bar{\kappa }}}$ as a convergence value of $\kappa _{n}$ below

23 $$\begin{aligned} \kappa _{n} = \frac{a(a+b) Z_{1} {\widehat{K}}_1(\alpha _{n,i}^2,\phi )}{2b} \end{aligned}$$

for $$i=2,4,6,17$$.

#### Estimation of $\alpha ^2$

We estimate $\alpha ^2$ as a convergence value of $\alpha ^2_{n,i}$ for $i=2,4,6,17$ which is defined as

24 $$\begin{aligned} \alpha ^2_{n,i} = \frac{A_{n,i} - \sin ^2\phi }{\cos ^2\phi } \end{aligned}$$

Here, $A_{n,i}$ are defined as below ($i=2,4,6,17$):

- Using $Z_2$. $A_{n,2}$ is a solution of the following 4th degree polynomial.
  25 $$\begin{aligned}  P^4x^4+(-4P^2+2P^4R)x^3+(-6P^2R+P^4R^2)x^2-2P^2R^2+R^2 \end{aligned}$$
  where $$R := 2ab+b^2$$ and $$P:=\frac{Z_2{\widehat{K}}_2(\alpha _{n-1,2}^2,\phi )}{\kappa _{n-1}}-\frac{1}{a}+\frac{1}{a+b}$$.
- Using $$Z_4$$.
  26 $$\begin{aligned}  A_{n,4} = \frac{Q_{n-1}-(a+b)^2}{s^2} \end{aligned}$$
  where $$Q_{n-1} := \left( \frac{2a\kappa _{n-1}}{aZ_{4}{\widehat{K}}_4(\alpha _{n-1,4}^2,\phi )-2\kappa _{n-1}} \right) ^2$$
- Using $$Z_6$$.
  27 $$\begin{aligned}  A_{n,6} = \frac{Q_{n-1} - a^2}{s^2} \end{aligned}$$
  where $$Q_{n-1} := \left( \frac{2a(a+b)\kappa _{n-1}}{(a+b)Z_{6}{\widehat{K}}_6(\alpha _{n-1,6}^2,\phi )+2\kappa _{n-1}}\right) ^2$$
- Using $$Z_{17}$$.
  28 $$\begin{aligned}  A_{n,17} = \left( \frac{2\kappa _{n-1}}{asZ_{17} {\widehat{K}}_{17}(\alpha _{n-1,17}^2,\phi ) + 2\kappa _{n-1}}\right) ^2 \end{aligned}$$

#### Inverse Method III: $Z_1$, $Z_{1}^{\perp }$, $Z_i$, and $Z_{i}^\perp$ are measured in $\theta =0$, $\varphi =0$ for $i \in \left\{ 2,4,6,17\right\}$

Assumption 1.: Same as method I
Assumption 2.: Unknown $$\phi (\ne 0)$$

**Figure 7:**
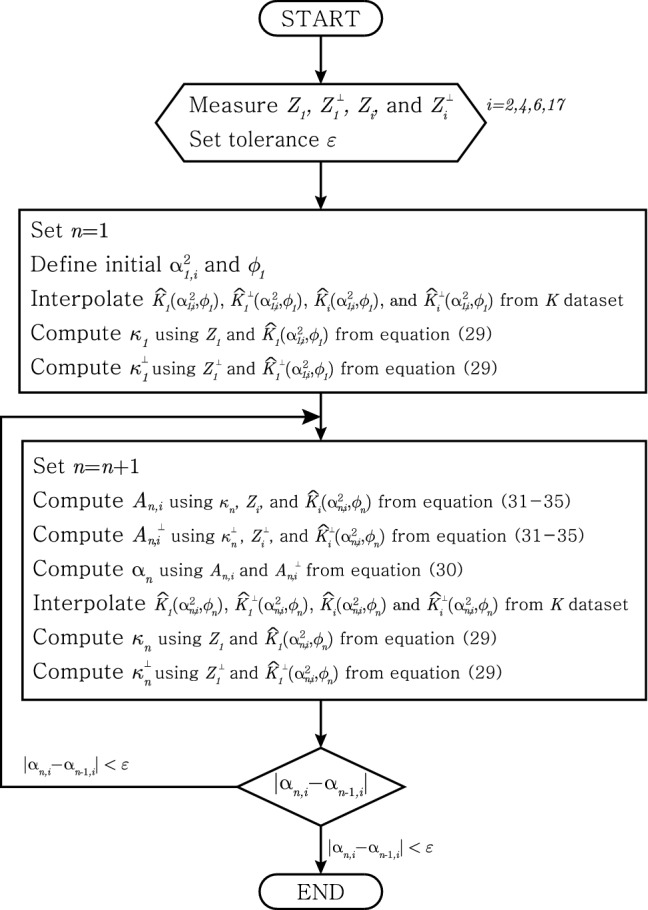
Iteration scheme of the method III.

Method III, which does not require to know $\phi$, is most practical method in this paper. This method uses four electrodes on one side and two faces perpendicular to each other (e.g. [ES] and [SE] planes in Fig. 1) i.e. eight electrodes total. At first, we compute $\kappa _n$ ($\kappa _n^\perp$) using the $Z_1$ ($Z_1^\perp$) and $K_1$ ($K_1^\perp$) in each plane (perpendicular plane). From the $\kappa _n$ ($\kappa _n^\perp$) and the $Z_i$ ($Z_i^\perp$) and $K_i$ ($K_i^\perp$) in each plane, we compute *A* ($A^\perp$). Now, $\alpha _n$ is found using *A* and $A^\perp$. The loop is made by putting $\alpha _n$ into the $K_1$ and $K_i$ ($K_1^\perp$ and $K_i^\perp$). The flowchart can be seen in Fig. 7.

#### Estimation of ${{\bar{\kappa }}}$

We estimate ${\bar{\kappa }}$ from the convergence value of $\kappa _{n}$ and $\kappa _{n}^{\perp }$. Note here that if the iterative process converges well, then $\kappa _{n}$ and $\kappa _{n}^{\perp }$ converges to the same value which is ${{\bar{\kappa }}}$.

29 $$\begin{aligned} \kappa _{n} = \frac{Z_{1} {\widehat{K}}_{1}(\alpha _{n,i}^2,{\widehat{\phi }}) a(a+b)}{2b} \quad \text{ and }\quad \kappa _{n}^{\perp } = \frac{Z_{1}^{\perp } {\widehat{K}}_{1}^{\perp }(\alpha _{n,i}^2,{\widehat{\phi }}) a(a+b)}{2b} \end{aligned}$$

for $$i=2,4,6,17$$.

#### Estimation of $\alpha ^2$ and $\phi$

We estimate $\alpha ^2$ and $\phi$ as a convergence value of $\alpha _{n,i}^2$ and $\phi _{n,i}$, respectively, as *n* goes infinity which are defined by

30 $$\begin{aligned} \alpha ^2_{n,i} = A_{n,i} + A_{n,i}^{\perp } -1 \quad \text{ and }\quad \phi _{n,i} = \frac{1}{2} \arccos \left( \frac{2A_{n,i} -1 -\alpha ^2_{n,i}}{\alpha ^2_{n,i}-1}\right) \end{aligned}$$

for $i=2,4,6,17$. We define $A_{n,i}$ and $A_{n,i}^{\perp }$ as below:

- Using $Z_2$ and $Z_2^\perp$. $A_{n,2}$ and $A_{n,2}^{\perp }$ can be found by solving the below 4th degree polynomial.
  31 $$\begin{aligned}  P^4x^4+(-4P^2+2P^4R)x^3+(-6P^2R+P^4R^2)x^2-2P^2R^2+R^2 \end{aligned}$$
  where $R := 2ab+b^2$ and *P* is defined as below:
  32 $$\begin{aligned}  P := \left\{ \begin{array}{ll} \frac{Z_2 {\widehat{K}}_{2}(\alpha _{n,2}^2,{\widehat{\phi }}) }{\kappa } -\frac{1}{a}+\frac{1}{a+b} &{} \text{ is } \text{ to } \text{ find } A_{n,2} \\ \frac{Z_2^\perp {\widehat{K}}_{2}^\perp (\alpha _{n,2}^2,{\widehat{\phi }})}{\kappa }-\frac{1}{a} +\frac{1}{a+b} &{} \text{ is } \text{ to } \text{ find } A_{n,2}^\perp \end{array}\right. \end{aligned}$$
- Using $$Z_4$$ and $$Z_4^\perp $$.
  33 $$\begin{aligned}  A_{n,4} = \frac{Q_{n-1}-(a+b)^2}{s^2},~~ A_{n,4}^{\perp } = \frac{Q_{n-1}^{\perp }-(a+b)^2}{s^2} \end{aligned}$$
  where $$Q_{n-1} := \left( \frac{2a\kappa _{n-1}}{aZ_{4} {\widehat{K}}_{4} (\alpha _{n,4}^2,{\widehat{\phi }})-2\kappa _{n-1}} \right) ^2$$ and $$Q_{n-1}^{\perp } := \left( \frac{2a\kappa _{n-1}}{aZ_{4}^{\perp }{\widehat{K}}_{4}^\perp (\alpha _{n,4}^2, {\widehat{\phi }})-2\kappa _{n-1}} \right) ^2$$.
- Using $$Z_6$$ and $$Z_6^\perp $$.
  34 $$\begin{aligned}  A_{n,6} = \frac{Q_{n-1} - a^2}{s^2},~~ A_{n,6}^{\perp } = \frac{Q_{n-1}^{\perp } - a^2}{s^2} \end{aligned}$$
  where $$Q_{n-1} := \left( \frac{2a(a+b)\kappa _{n-1}}{(a+b)Z_{6} {\widehat{K}}_{6} (\alpha _{n,6}^2,{\widehat{\phi }})+2\kappa _{n-1}}\right) ^2$$, $$Q_{n-1}^{\perp } := \left( \frac{2a(a+b)\kappa _{n-1}}{(a+b)Z_{6}^{\perp } {\widehat{K}}_{6}^\perp (\alpha _{n,6}^2,{\widehat{\phi }})+2\kappa _{n-1}}\right) ^2$$
- Using $$Z_{17}$$ and $$Z_{17}^\perp $$.
  35 $$\begin{aligned}  & A_{n,17} = \left( \frac{2a\kappa _{n-1}}{s(aZ_{17} {\widehat{K}}_{17} (\alpha _{n,17}^2,{\widehat{\phi }})+2\kappa _{n-1})}\right) ^2,~~\\& A_{n,17}^{\perp } = \left( \frac{2a\kappa _{n-1}}{s(aZ_{17}^{\perp }{\widehat{K}}_{17}^\perp (\alpha _{n,17}^2, {\widehat{\phi }})+2\kappa _{n-1})}\right) ^2 \end{aligned}$$

### Numerical simulations

The numerical experiments are performed using Comsol (Comsol Multiphysics, Inc., Burlington, MA) and Matlab (The Mathworks, Inc., Natick, MA). In order to simulate needle muscle impedance data, a box in Comsol with $40\times 40\times 40$ ($\hbox {cm}^3$) is created. The needle dimensions are length 20 (cm), width 2.15 (cm), and thickness 0.2 (cm). The needle is inserted vertically into the domain, i.e. $\phi = 0$, $\varphi = 0$ while $\theta$ is 0 for method I and $\pi /6$ (radian) for method II and III. The needle electrodes have radius 0.05 (cm) with distances $a_0=1$ (cm), $a=1$ (cm), $b=1$ (cm), $c=0.5455$ (cm), $s=0.9893$ (cm) (details in Fig. 1 b). To estimate the anisotropic permittivity of the domain, a sinusoidal current of 1 (mA) was injected from 1 kHz to 1 MHz. Experimental results verifying ‘Convergence’, ‘Sensitivity’ and ‘Robustness’ were performed at 1 kHz. The used transverse electrical conductivity $\sigma _{\mathrm{T}}$ is from 0.341 to 0.503 (S/m) and relative permittivity $\varepsilon _{\mathrm{r,T}}$ is from $2.59\cdot 10^4$ to $1.84\cdot 10^3$ (dimensionless) from skeletal muscle at specified frequencies^35,36^. Longitudinal (denoted by the subscript L) permittivity values were computed from transverse permittivity values using a (constant) anisotropy ratio $\alpha ^2=0.4$, unless otherwise noted. Methods I and II use needle impedance values $Z_1$ and $Z_6$ while method III uses $Z_1$, $Z_6$, $Z^{\perp }_1$, and $Z^{\perp }_6$. Simulated impedances in method I are obtained assuming $\phi =0$, whereas method II and III assume $\phi =\pi /6$. Here, the angle $\phi =\pi /6$ for method II and III is chosen to satisfy the assumptions of the methods which is $\phi \ne 0$. Note that, in method II and III, $\phi$ may select any angle satisfying $\phi \ne 0$, which is the assumption of the method II and III. For the iteration process, the initial anisotropy ratio $\alpha ^2$ is set to 1 and consequently initial $K(\alpha ^2,\phi )$ is obtained with $\alpha ^2=1$.

## Results

Numerical experiments were performed to evaluate three iterative reconstruction methods: (i), method I (corresponding equations are 17 - 21); (ii), method II (corresponding equations are 22, 23); (iii), method III (corresponding equations are 24, 25). The results confirm the convergence of the methods, their sensitivity to the presence of measurement noise, and their robustness to experimental positioning errors through numerical experiments. In addition, the three proposed methods were applied to estimate the electrical properties of the muscle, conductivity and relative permittivity, in the frequency range of 10 kHz to 1 MHz. The cross-shaped needle in method I has its major axis aligned with respect to the *z*-axis and the [ES] face is the *yz*-plane, i.e. $\theta =0$, $\phi =0$, $\varphi =0$ (Fig. 2). Method I is then extended to method II by considering needle’s rotation angle $\phi \ne 0$. In this numerical simulation, we assume $\phi =\pi /6$ with respect to its major axis. For method III, the angle $\phi$ is assumed to be unknown. The two impedances $z_1$ and $z_6$ are used in both method I and method II. In method III, four impedances $z_1$, $z_1^\perp$, $z_6$ and $z_6^\perp$ are used to estimate muscle permittivity values.

**Figure 8:**
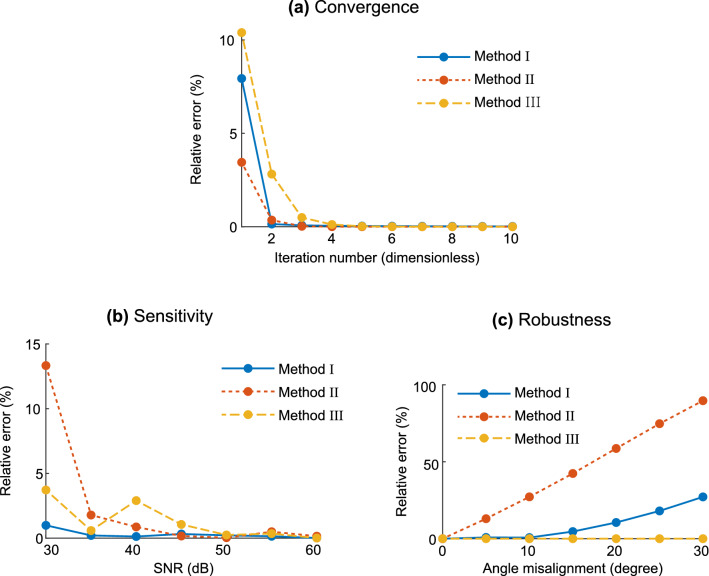
Performance comparison between methods considering the estimated anisotropy ratio $\alpha ^2$. **(a)** Convergence of the with iteration number. **(b)** Sensitivity to measurement noise. **(c)** Robustness in front of needle angle misalignments.

To compare the three methods, the following three observations were made; (1) ‘Convergence’: result error according to the number of iterations to which the method is applied (Fig. 8a), (2) ‘sensitivity’: result error according to the noise level (Fig. 8b), (3) ‘Robustness’: result error according to angle error (Fig. 8c). Method I was developed to be applied when the needle is inserted perpendicular to the muscle and one side of the needle is parallel to the direction of the muscle fiber. According to the experimental results, we recommend to use method I to obtain stable results at all SNRs, but to obtain accurate results, the angular misalignment with respect to the anisotropy direction should be less than 10 degrees. Method II was developed to be applied when the needle is inserted perpendicularly to the muscle, and the angle between one side of the needle, the direction of the muscle fiber is not 0 and the angle is known. According to the experimental results, method II should be used only when an SNR of 40 dB or more and a very accurate angular mismatch are guaranteed. Method III was developed to be applied when the needle is inserted perpendicular to the muscle. It can be applied without knowledge of muscle fiber orientation. Experimental results show that method III provides the most stable and accurate results when an SNR of 50 dB or higher is guaranteed.

### Convergence

As mentioned above, the methods presented are iterative. Therefore, here we evaluate their convergence to the true permittivity value. We quantified the error of the resistivity anisotropy ratio $\alpha ^2$ as a function of the iteration number. Figure 8a shows all three methods proposed converge to the true value and the relative error is lower than 0.1% after five iterations. All three methods showed good convergence in which the error decreased as the number of iteration increased in common. If the number of iteration was less than 5 times, method II was the most accurate and method III was the least accurate, but if the number of repetitions was more than 5 times, all three methods had a very small error of close to 0%.

### Sensitivity

Figure 8b shows the error of the estimated anisotropy ratio $\alpha ^2$ under the presence of random noise varying the SNR from 30 to 60 dB. Overall, these results show the estimated parameter is not sensitive to noise if the SNR is larger than 35 dB, with a relative error lower than 5%. The SNR is computed as

36 $$\begin{aligned} \text{ SNR } = 10\log _{10}\left( \frac{\text{ impedance } \text{ value }}{\text{ standard } \text{ deviation }}\right) ^2 \end{aligned}$$

From the given SNR range (30 to 60 dB), the simulated noisy impedance is generated with additive random noise sampled from standard Gaussian distribution with appropriate standard deviation. Methods I and III clearly showed a tendency for errors to decrease as SNR increased. Method II showed that as the SNR increased as a whole, the error tended to decrease, but when the SNR was 40–45 dB, an unexpected large error occurs. Since the measurement noise is randomly made, a large error may occur in the 40–45 dB SNR by chance. As a result of performing a number of random measurement noise at 30–60 dB, errors tend to decrease as SNR increases overall, but errors are large even in certain SNRs.

### Robustness

Figure 8c shows the reconstruction error of the estimated anisotropy ratio $\alpha ^2$ assuming an experimental misalignment of the cross-shaped needle once inserted into the muscle. Out of the three methods developed and tested, method III is the most robust to angle errors. Methods I and II are very weak against angle misalignment, in other words, the larger the angle mismatch, the greater the error. Method II is more vulnerable to angle misalignment than method I. On the other hand, method III is robust against angle misalignment, because it does not require any prior information about the angle. In this experiment, an error of less than 0.1% was shown in the case of angular misalignment.

### Reconstruction of anisotropic muscle electrical properties

**Figure 9:**
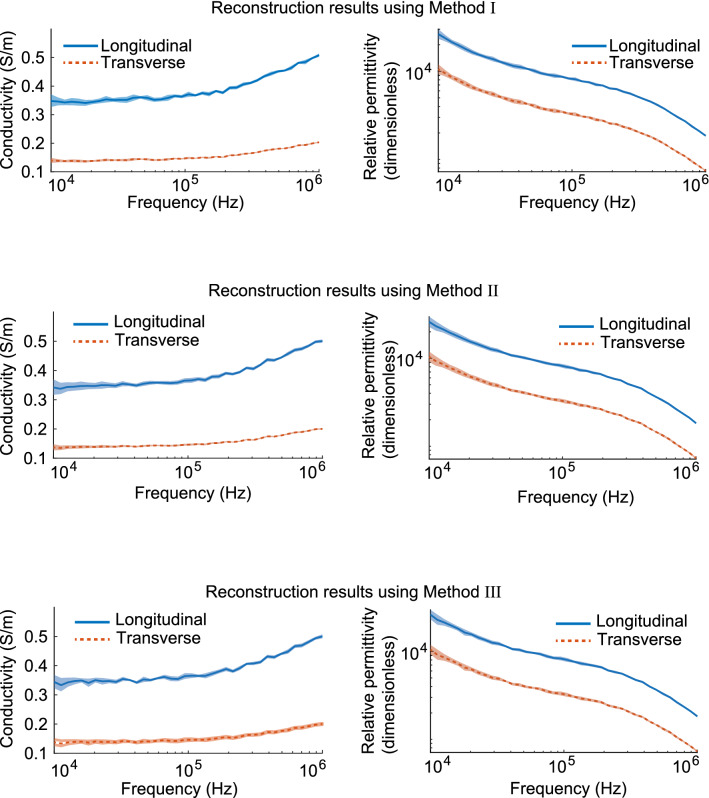
Estimated anisotropic permittivity (i.e., conductivity and relative permittivity) using method I, II, and III. The solid lines represent the true values, whereas the shaded area represent $\pm 3$ times the standard deviation based on the estimated value.

Figure 9 shows the results of estimating the conductivity and relative permittivity (electrical properties) of anisotropic muscles for frequencies using each of the three methods; method I (Eq. 18), (Eq. 21) with $Z_1$ and $Z_6$, method II (23), (Eq. 24) with $Z_1$ and $Z_6$, and method III (Eq. 29), (Eq. 30) with $Z_1$, $Z_1^\perp$, $Z_6$ and $Z_6^\perp$. In this numerical experiment, when the angle misalignment was 1 degree and the noise SNR was 30 dB, the average and standard deviation of the estimated values were used by applying each method 10 times. The solid and dotted lines show true muscle electrical properties (EP)^35,36^ and transparent areas represent $\pm 3$ times the standard deviation (99.7% confidence interval) based on the estimated value from method I, II, and III. For all three methods, the true values were within the 99.7% confidence interval. The 99.7% confidence intervals for longitudinal and transverse EPs were small enough to distinguish between longitudinal and transverse EPs.

## Discussion

This modeling study demonstrates the potential for using a novel cross-shaped needle for the assessment of two-dimensional anisotropic muscle permittivity property. While the technology itself still requires actual development, its potential for simplifying assessment of this electrical tissue property could be valuable. Specifically, by better understanding tissue permittivity, it could become possible to more accurately assess and understand alterations in muscle tissue in certain diseases and their impact on standard electrophysiological testing^25^. For example, by applying this needle and analyzing the resulting data, it would become possible to better understand the origins of the morphology of motor unit potentials recorded at a distance from the source generator (i.e., the cumulative depolarizing myofiber membranes)^26^. Similarly, we would be able to better understand the limits of detection of smaller discharges, such as fibrillation potentials, as one moves farther from the source. Indeed, these very basic concepts have been minimally studied to date and, in these authors’ view, remain relatively poorly understood^6^. Detailed work in this area could greatly expand the basic tenets underlying the field of clinical neurophysiology.

Beyond these new insights into the underpinnings of acquired needle EMG data, the ability to measure tissue permittivity with a single needle could also have important direct clinical value^27,28^. To date, needle impedance-related works require multiple simultaneous 4-electrode needle insertions to measure tissue permittivity^20,21^. The invasiveness of these approaches limit their clinical translation, but this could be obviated with the construction and implementation of the needle design proposed here^29^. Being able to measure the permittivity directly with a single needle insertion could open up an entirely new area of research with direct clinical application, including using these actual tissue property to assist in neuromuscular diagnosis, and even more importantly, to assess subtle effects of therapeutic intervention on muscle health.

There are several limitations to this study and our proposed needle design. First, this is only a modeling study and a number of both theoretical and practical simplifications have been made, including the assumption of relative homogeneity of muscle tissue. However, many NMDs are patchy with some regions of a given muscle being severely affected while other regions are entirely normal or only minimally affected^30,31^. While we have made first inroads into modeling such heterogeneities^32^, this would have made our analysis here difficult and was not attempted here. Second, muscle conditions are not static and thus, it is possible that there may be changes in tissue permittivity making it difficult to align EMG data obtained even if there is a relatively short time lag between application of the cross-needle and the insertion of a standard concentric EMG needle in the same region. The third related concern is that this cross-needle itself might injure the muscle thus potentially altering impedance readings. Standard EMG needles are round for many reasons, a major one of which is to ensure patient comfort (as well, as of course, simplicity in design and manufacture). The needle designed here, although pointed, to some extent represents two orthogonal blades being inserted into the muscle. Even if relatively small, it would like still injury muscle and cause localized bleeding which in itself could negatively impact and distort the measured permittivity. It is worthwhile to note, however, that other large needles do exist and are used routinely for muscle assessment such as the Bergstrom biopsy needle, which actually typically requires a small incision in the overlying skin to place appropriately in the muscle. Of course, it may be theoretically possible to make this cross-needle very small (e.g., approximately 26 gauge, based on a circle circumscribing the cross), and thus keeping any tissue damage quite limited. A fourth concern is that, while we have included anisotropy in our analyses, as well as a study of needle misalignment, muscle fiber orientation can be complex, and thus this modeling remains a vast simplification of any observed data. Finally, muscle is is a dynamic organ and in these analyses we have considered only the muscle at rest. However, in order to generate actual contraction of the muscle, motor unit potentials is required. Even an isometric contraction would still likely alter many aspects of muscle structure, including producing slight shifts in muscle fiber orientation (and hence anisotropy), altering the relative extracellular/intracellular volume ratios, and impacting muscle blood volume, a feature that was not even included in this model.

A single surface flat electrode can only be used in Methods I and II. In order to apply Method III, it is required to have a surface and its perpendicular surface. Accordingly, the minimum requirement is L-shaped needle electrode which has two planes perpendicular to each other. However, the reason why we proposed a cross-shaped needle electrode rather than an L-shaped needle electrode is because of (1) structural stability resulting from the symmetry of the shape, and (2) the efficiency of measuring four regions with one insertion.

In conclusion, we have performed a modeling study on a novel impedance-measuring needle that has the potential of providing robust assessments of muscle volume conduction properties. To move these innovative concepts forward, it will next be necessary to design and produce a working version of this needle electrode, likely for initial application and study in larger NMD animal models. This work is now being planned.
